# Increasing the focus on critical appraisal of trials and financial conflict of interest in medical training programs: a perspective from oncology

**DOI:** 10.3389/fmed.2025.1582732

**Published:** 2025-10-08

**Authors:** Ashley J. Duits, Michael J. Samson, Rijk O. B. Gans, Izzy Gerstenbluth, Jamiu O. Busari, John-John B. Schnog

**Affiliations:** ^1^Institute for Medical Education, University Medical Center Groningen, Groningen, Netherlands; ^2^Curaçao Medical Center, Willemstad, Curaçao; ^3^Curaçao Biomedical and Health Research Institute, Willemstad, Curaçao; ^4^School of Health Professions Education, Maastricht University, Maastricht, Netherlands

**Keywords:** critical appraisal of medical trials, financial conflict of interest, value based health care (VBHC), hidden curriculum, agents of change, transformative medical education, medical education moonshot program

## Abstract

Improving medical education programs is key for achieving true value-based healthcare. Worldwide several approaches have been proposed for adapting current medical curricula in order to better foster modern medical professionals as agents of change. Adaptations of (mostly) competency-based curricula include improved attention for topics like public health, social determinants of health, inclusivity and social justice. In this article the authors argue, using an oncology perspective, for inclusion of two key topics in order to ensure that improved medical curricula foster critical consciousness and are transformative. The authors describe the importance of adding critical trial appraisal and awareness of the challenges of financial conflict of interest (FCI) in medical decision making throughout the curriculum. In the field of oncology (as in other medical areas) approval and uptake in treatment guidelines of costly drugs are regularly based on methodologically flawed clinical trials. Moreover, in an already financially strained environment these low-value treatments further impede countries of better supporting only high-value treatment and prevention programs. The authors show that FCI negatively impacts drug approval processes, journal publications, guideline inclusion and patient advocacy group activities. In this scholarly perspective the authors strongly argue for including these topics in modern curricula with due attention for the necessary cultural change of faculty and clinical learning environment. An educational moonshot program based on critical pedagogy is advocated in which both High Income Countries (HIC) and Low- and Middle-Income Countries (LMIC) contribute to achieve the necessary transformative medical education programs necessary for the required agents of change.

## Oncological care and the cancer moonshot program

Cancer causes 1 in 5 of deaths due to non-communicable diseases and poses both a global public health and economic challenge. The rising costs of contemporary oncological treatments are unsustainable even in affluent countries, whilst classical lifesaving chemotherapy drugs are not universally available in low- and middle-income countries (LMICs) and some high-income countries (HIC) (1, 2).

The Cancer Moonshot program (a United States government supported initiative to reduce cancer burden) that fosters international collaboration, focuses on cancer’s health and economic impact on patients and strives for health equity in LMICs, has received renewed attention (3). The program aims to introduce effective management tools such as better symptom management, effective medical treatments, and early cancer detection. Also, research is supported to develop novel and equitable therapies. To achieve its goals and have an impact on cancer, investing in knowledge translation programs is important.

There is reason to believe that focusing solely on knowledge translation and the goals of effective management tools are likely to benefit only a small proportion of patients. Hence, caution must be exercised when considering the effectiveness of such medical treatments in HIC such that marginalized groups and the weak in society are not mis- or underrepresented. The often-limited efficacy (i.e., the benefit of an experimental treatment within a randomized controlled trial (RCT)) of new oncological treatments mostly does not translate to increased effectiveness (i.e., the benefit of a treatment in real life of patients). Shortcomings in design, execution, and interpretation often characterize contemporary oncological trials of new therapeutic interventions. However, the low bar for approvals of major drug regulatory agencies such as the Food and Drug Administration (FDA) and European Medicines Agency (EMA), result in approvals of such questionable treatments with subsequent practice guideline uptake (4). As a result, many high-cost interventions with limited or no benefit have saturated the market (4, 5). Against this backdrop, the Lancet Commission on Cancer and Health Care Systems strives to improve cancer control equitably. It clearly emphasizes the necessity for more attention to overdiagnosis, overtreatment, and escalating costs for treatments void of better outcomes. The HIC treatment programs should not automatically be considered the standard for effective global cancer control (6). Recognizing this fact is critical for any translational program to safeguard value-based care (a framework for maximizing the value of care for patients whilst reducing costs) and high-value interventions in both HIC and especially in LMICs with limited economic resources.

For that reason, medical professionals should be competent to critically evaluate the true value of care (or a treatment), defined as the costs of an intervention and the benefit for the patient of any available treatment (7). Even though there is the assumption that physicians are sufficiently educated to evaluate whether a medical intervention is both efficacious and effective, empirical studies have shown that physicians generally overestimate benefit and underestimate harm across a broad range of medical interventions (8).

We recently addressed the need to raise the bar for conducting oncological clinical trials and drug approvals (4). One of our recommendations, as also emphasized by others (5, 9), was the reevaluation of current medical education programs for physicians (and other health professionals) to better appraise the limitations of evidence generated by current clinical trials.

In this scholarly perspective, we argue that existing medical educational programs, mostly competency-based ones (10), should be adapted to include essential topics for physicians to become agents of change, i.e., people who can truly bring about the required change necessary to achieve value-based healthcare (11).

Transformative learning programs based on critical pedagogy (a philosophy of education enabling and encouraging critical thinking) that foster critical consciousness (recognizing, challenging and acting for solving oppressive social structures) and include ongoing medical ethics education and leadership development are essential for training young medical professionals to become agents of change (11, 12). Improvement of students’ and health professionals’ critical interpretation skills of clinical trial data should be prioritized and embedded in curricula that are better designed for achieving awareness and understanding of the determinants of health (13), social justice and how their future role as physicians can impact not only individual patients but their community as well (12, 14).

Importantly, an ongoing in-depth focus on medical ethics, including increased focus on financial conflicts of interest (FCI) potentially influencing medical decision-making, should be a common thread throughout the entire curriculum (4, 15).

## Increased focus on critical clinical trial evaluation and FCI in medical education programs

Current competency-based medical education frameworks such as Accreditation Council for Graduate Medical Education (ACGME) (16) and Canadian Medical Education Directives for Specialists (CANMEDS) (10) do not explicitly address value-based healthcare in medical curricula. Hence, several programs have been introduced, such as Health Systems Sciences (HSS) (17), to include patient and population health, healthcare policy, medical ethics, and value-based healthcare into medical curricula. This is in addition to the existing basic and clinical science programs in the undergraduate and graduate curriculum (18–20). Despite these changes, unfortunately, certain key skills/competencies remain underserved.

These include competencies needed to critically evaluate trial design, outcome, validity, and applicability in evidence-based medicine (EBM) and clinical practice guidelines.

We propose that current training programs should specifically include content to enable the development of these skills (21–23).

### Critical appraisal of clinical trials is insufficiently taught

Critical appraisal of clinical trials is the systematic and careful analysis of trials to judge its true value and relevance for use in EBM (24).

Several studies have underscored the importance of implementing teaching modules for critical appraisal of clinical trials (25, 26). Presently, different systems for general evaluation of the quality of clinical trials (23, 24) are in use albeit their scope is limited to technical aspects (e.g., methods of randomization), foregoing focus on broader aspects of trial quality and applicability (e.g., was the control arm the standard of care, was cross-over correctly applied). A technically soundly performed RCT can still be useless when, for example, the primary endpoint is an unvalidated surrogate endpoint that does not measure what truly matters to patients (overall survival and/or quality of life) (27). A more hands-on broad approach, as widely made available by experts in the field and that is easily accessible for students and physicians should be embedded in clerkship-, residency and fellowship programs.^1^

### FCI undermines clinical judgement

FCI exists when medical decision making is influenced by financial gain (28). Curricula designers and medical professionals involved in designing training programs on critical appraisal of research trials and EBM should focus on upholding the code of medical ethics (e.g., the AMA code of Medical Ethics^2^ and at the same time ensure that there are medical ethics training programs) (29) to foster critical reasoning. These programs should include developing ethical appraisal for recognizing and addressing potential FCI faced by medical professionals in patient care, scholar and research activities (e.g., GMC professional standards 2024.^3^ FCI with pharmaceutical industries has been associated with decision-making at many different levels like drug and device research and development and the associated regulatory processes. FCI associations with the quality of output in peer-reviewed journals, scientific meetings output, uptake in clinical practice guidelines (30), websites and social media contents and patient-advocacy organizations activities (31–36) have also been reported. At each of these levels, FCI has been shown to bias the appraisal of novel and often costly interventions in a way that low-value interventions based on clinical trials with important shortcomings are still approved and incorporated into treatment guidelines (15, 37, 38). FCI is clearly associated with prescribing of both low-value interventions and brand-name drugs (39, 40). For addressing social accountability, fostering of efficient use of healthcare resources is critical (41). Therefore, a fundamental understanding of the negative impact of FCI by medical doctors/experts in the current drug- and device development landscape and its consequences for individual patient care, societal health care spending and value- based care must be addressed early in the curricula. This is needed irrespective of whether a trainee’s intent is to pursue an academic research-focused career or a career primarily as a care provider (42, 43). In a web-based survey in France of 2,101 medical students 85.2% of the students reported being inadequately educated about FCI (44). In a comparable Korean study of 388 medical students 63.7% reported insufficient FCI-education supporting the need for improved FCI-education in medical curricula (45).

## A critical pedagogy approach

As extensively described by Frenk et al. (11), education programs need to include developing expertise in resource management, promoting evidence-based policies (including treatment policies and guidelines), and the critical assessment of existing policies. Furthermore, we argue that fundamental global health and public health themes, including sections dedicated to determinants of health (46, 47) should be included to ensure the development of the necessary professional sense of social accountability (46, 48, 49). Importantly, such programs should promote social justice and address existing health inequities (50). Making use of critical pedagogy (14) as an educational framework to design such programs we believe would ensure that these topics are well embedded in the curriculum. This should then also offer the opportunity for training programs to ultimately avoid just supporting the status quo (described as “banking education: students only repeating educator provided information” (12)) and instead help achieve the required changes. Changes necessary not only in health care but, more importantly, toward achieving equitable conditions to attain healthier lives.

These competencies are all necessary for effectively evaluating (new) treatment options and addressing the shortcomings of current drug evaluation and approval procedures by regulatory agencies (4, 11). This is corroborated by the recent WHO report that clearly underlines the importance of including these topics in medical education programs (51). Fittingly, such an educational approach aligns with the currently held view by healthcare students that preventive medicine and public health are fundamental aspects of their role as future health professionals (52). In a recent survey of 2,212 students from 91 countries 80% of the students describe public health management and focusing on preventive health as crucial priorities (53).

## Achieving cultural change: faculty development and addressing the hidden curriculum

### A renewed approach to faculty development

A transformative learning approach based on the concepts of critical pedagogy and fostering the necessary critical consciousness (12, 14) is necessary to enable medical students to understand the principle of value-based care and become true “agents of change.” A much-needed renewed approach to faculty development would require culture change that includes the critical evaluation of existing norms and values and how to effectively cope with potential emergent non-customary roles and attitudes (54).

A recent publication (55) addressed the most important ideas and challenges perceived by health educators during the re-alignment of curricula. For the renewal of a program, a clear institutionally formulated mission and vision statement is needed. There also has to be a demonstrable urgency for change, change-management strategy, and leadership skills development (41). In our view the program should enable change by offering practice-focused support, promoting the creation of communities of practice for learning (56), engagement and networking, and providing guidelines for addressing critical consciousness where needed for the available staff. It is important that faculty developers expertly guide this potentially disruptive process by providing opportunities for dialogue and reflection over time to maintain these critical competencies.

### Addressing the challenges of the hidden curriculum

To attain a medical curriculum that effectively trains students in value-based care, a cultural change in the clinical environment would be necessary. For programs to be effective authentic experiential learning environments that regularly address critical clinical trial evaluation and FCI will be needed. The hidden curriculum in most clinical learning environments influences the professional development of trainees through everyday experiences of habits, customs, perspectives, and vocabulary they encounter (57). The potential dissonance the hidden curriculum presents to trainees needs to be assessed and understood. Special attention should be given to the impact of preceptors’ role modeling on students and trainees. Specific strategies should be evaluated with the input of clinical teachers (58) to bridge the existing knowledge and attitude gap effectively and (if necessary) reform the learning environment (e.g., by introducing targeted faculty development courses) (59). Medical school research programs should be in line with the above-described cultural change.

Box 1 and Table 1 describe practical steps undertaken to include critical appraisal of trials and FCI in clinical clerkships, resident programs and continuous medical education provided at the Curaçao Medical Center.

**Table 1:** Example of the tool to measure/analyze the entrustible professional activity (EPA) sensible care for medical students.

EPA sensible care oncology - 4 weeks internship
Start rotation: *Receive literature.* Schnog JB, Samson MJ, Gans ROB, Duits AJ. An urgent call to raise the bar in oncology. Br J Cancer 2021;125:1477–85. The intern needs to choose an interesting case (together with the supervisor) within the first 2 weeks in which a treatment decision needs to be critically evaluated and valued. *Lecture on evidence* (‘How do we know what we know and why do we do what we do?’). Topics to cover: Aims of treatment. Endpoints of trials. Different types of clinical studies and why we should base our guidelines on well designed and well executed phase 3 RCT’s (focus on bias). End of week 2: *Receive a case preferably from a ‘real’ patient* (e.g., patient with resected NSCLC, prescribe adjuvant Osimertinib? Preferably, a case in which the intern was involved in his/her care). Receive a published clinical trial pertaining to the case received (e.g., Tsuboi M et al. Overall survival with Osimertinib in resected EGFR-mutated NSCLC. N Negl J Med 2023;389:137–47). *Lecture on example of a RCT in oncology* (‘Caveats of contemporary oncological RCT’s). Topics to cover: Patient characteristics. In- and exclusion criteria Adverse events Different clinical trial endpoints. Control arm problems (physician choice, standard of care, post-protocol treatment, crossover). Financial conflict of interest (FCI) End of week 4: Intern presents the case (4–5 slides), accompanying RCT and discusses; would the intern treat or not? The presentation, interpretation and if applicable extra literature will be discussed with and prepared together with the supervisor. Intern formulates own interpretation and arguments how he/she values the clinical decision-making coming forth from the case and accompanying RCT. The intern can: Clearly formulate the research question at hand. Formulate his/her interpretation of the evidence belying the clinical question. Provides his/her interpretation of the intervention in relation to the impact thereof on society. Intern completes an analysis – using the tool below and undergoes assessment according to the Entrustable Professional Activity (EPA) *Levels of the EPA* Observation: At this level, the intern primarily observes the analysis/tool being performed by a more experienced colleague. They may have limited or no hands-on involvement in the activity. Assistance: The trainee is involved but requires direct supervision and guidance from a more experienced colleague. They may assist in various aspects of the task but are not yet capable of performing the analysis/using the tool independently. Supervised Performance: The trainee can perform the analysis with some level of independence but still requires supervision and feedback from a supervisor. They may be able to complete most aspects of the task on their own but may need assistance with more complex or challenging components. Unsupervised Performance: At this level, the intern is capable of independently performing the activity without direct supervision. They have demonstrated competence and can be trusted to carry out the task without oversight. Teaching or Supervising Others: In some frameworks, this level may be included to indicate that the trainee has reached a level of proficiency where they can teach or supervise others in performing the activity. This indicates a higher level of mastery and expertise.
1. Critical appraisal 101
On what class of research is the treatment recommendation/guideline based? *(understand key difference between phase 3 RCT and observational studies, retrospective studies, case–control studies, phase 2 RCT etc.)*.
Understand when phase 3 RCT are preferred for assessing efficacy of an intervention.
Understand difference of activity, efficacy and effectiveness.
Assessing the phase 3 RCT:
What did they do?
Who did the study?
To whom did they do it?
How did they do it?
Was the control arm appropriate?
What primary endpoint appropriate?
Was the primary endpoint achieved?
What was the magnitude of effect?
Was time toxicity accounted for?
If applicable, was post-protocol therapy adequate?
Was cross-over appropriately ensured or prohibited?
Social accountability
What are the costs of the proposed treatment per patient? *(Take into account drug price, cumulative price for treatment* (e.g., admission), days of work etc.).
What is the impact of applying this treatment for care delivery for oncology patients?
What is the societal impact of applying this treatment?
Calculate the cost per averted event.
Should this treatment, in your opinion, be offered to this particular patient and/or in general? The intern presents his or her arguments to treat or not to treat, and formulate an opinion on potential down-stream consequences.
Ecosystem of developing, bringing to market and guideline uptake of new treatments and diagnostics.
What are stages in drug development (laboratory, animal studies, phase 1–3 [registration trials]).
How are phase 3 RCT trials in oncology funded?
Who performs, analyses, and writes registration trials?
Describe influence of the pharmaceutical industry.
Describe width of financial conflict of interest.
Where do oncology reviewers work after the FDA?
What happens after a drug is approved by the FDA?
EMA and other HTA follow.
Are authors of clinical guidelines independent?
FCI breadth in guidelines.
What is your opinion regarding FCI with pharmaceutical companies in relation to drug development, approvals guideline uptake?
Is the price of modern oncology treatments justifiable?
Describe relation between novelty, drug development, activity, efficacy and price.

RCT, Randomized controlled trial; NSCLC, non-small cell lung cancer; FDA, Food and drug administration; EMA, European Medicines Agency; FCI, Financial conflict of interest; EPA, entrustable professional activity.

BOX 1 **Experiential learning in value-based health clinical programs at the Curaçao Medical Center (CMC).**

As an affiliate teaching hospital of the University Medical Center Groningen (UMCG) CMC provides clinical clerkship programs for the final 2 years of the CANMEDS based UMCG undergraduate medical curriculum (60).

The clerkship program includes a professional behavior development course for developing reflective skills based on a mentoring portfolio with weekly guided intervision peer meetings and individual sessions (61, 62). Additionally, a 3-week value-based care training session is provided to all undergraduate and graduate students and trainees. This training includes how to critically evaluate clinical trials based on fundamental scientific principles and proper attention to potential financial conflict of interest (https://www.drugdevletter.com/p/how-to-read-and-interpret-a-cancer?r=tdvd8&utm_campaign=post&utm_medium=webPRASAD) (see Table 1).

## A medical education moonshot program for all countries

In addressing priorities for cancer research in LMIC, Pramesh et al. defined value-based care and economics as one of the priorities (63). However, support programs by HICs are mostly based on transplanting their current practices which are much less focused on value-based care and likely less desirable for LMICs. In view of the growing appreciation of the efficacy-effectiveness gap in oncology (64), the capacity for critical appraisal of evidence in relation to social justice, public health and determinants of health should be optimized early and continued throughout medical education programs. This core competence is key for adequate interpretation of the value of the myriad of ever increasing new potential treatments. This should help clinicians in protecting patients from low-value care and against both individual and societal financial toxicity, allowing (re)allocation of health care spending (65).

Fundamental understanding of the potential shortcomings of scientific output (4) by physicians is expected to guarantee critical evaluation of the relative value of and evidence for clinical interventions and the resulting quality of care, even when making use of improved evaluation frameworks provided by external scientific bodies (66, 67). Ultimately, medical professionals must have a better understanding and support for the policies of governing bodies such as the American Society of Clinical Oncology, on the importance of social determinants of health and health-related social needs for treatment outcome and trial design (68). The WHO recently stressed the importance of considering these issues in clinical trials in its latest guidance for best practices for clinical trials (69).

The non-alignment between the pharmaceutical industry, health regulation, and patient care has been extensively discussed, and potential solutions for this have been presented (70). However, after almost 20 years of various attempts to solve the issues, the situation remains the same or even seems to have worsened (64, 71). We believe that with a value-based care-driven approach, realignment can be achieved (4). Furthermore, the observations made for the field of oncology can be broadly applied to other fields in medicine. For example, a recent systematic review reported the lack of high-strength evidence for using surrogate markers as primary endpoints supporting FDA approvals for drugs in the treatment of non-oncologic chronic disease (72). The negative consequences of FCI in other medical fields is also widely recognized (28, 30, 73).

Therefore, investing in medical training programs that foster critical appraisal of trials and awareness of challenges of financial conflict of interest in medical decision making should be prioritized. Concomitantly steps should be taken to address the necessary faculty development and realignment of the hidden curriculum.

### A medical education moonshot program: a concerted HIC-LMIC effort

As a result of a medical education moonshot program covering all areas of medicine, well-trained medical professionals would promote only true high-value treatment programs and be the necessary agents of change for raising the bar. As part of a concerted effort by HIC and LMIC to develop and ensure value-based care in all countries, the introduction and support of radically improved medical education curricula would be of genuine benefit and should ultimately result in sustainable healthcare improvement. The recent COVID-19 pandemic and the ever-increasing financial strain on healthcare budgets in all countries, further aggravated, for example, by the current surge in the use of anti-obesity drugs (74, 75) has shown us the necessity of such an approach. Therein, critically evaluated and successful, cost-effective programs designed in LMIC could prove of great value in HIC (76). Several successful examples can be given of LMIC developed programs introduced in HIC like making use of Brazil’s family health strategy-based community healthcare workers (CHW) to provide household level support and advice during the COVID-19 pandemic (77). Currently these CHW are poised to play an important role in primary care and uptake of preventive services in the (deprived communities of the) UK (78). Another example is the well-documented positive impact of the Colombia-developed Kangaroo care practice (involving skin-to-skin contact between mothers and preterm-infants) on infant health that is routinely used in maternity wards worldwide (79).

As historically shown by the export of the Flexner Medical Education Model from the USA to medical schools abroad around 1917, medical education can be a successful tool in attaining healthcare improvement (11). Medical education would nowadays certainly benefit from a Global Health Reciprocal (GBHRI)- like approach for achieving innovative medical education programs in both LMIC and HIC countries (80) for attaining worldwide social accountable and value-based care (81). Excellent examples of such programs exist, such as the recently described critical pedagogy-inspired Brazilian medical education-based Pedagogy of Connection framework fostering students’ critical consciousness and more effectively connecting the student to social justice and compassion (82).

## Data Availability

The original contributions presented in the study are included in the article/supplementary material, further inquiries can be directed to the corresponding author.

## Footnotes

^1^ https://www.drugdevletter.com/p/how-to-read-and-interpret-a-cancer?r=tdvd8&utm_campaign=post&utm_medium=web

^2^ https://code-medical-ethics.ama-assn.org

^3^ https://www.gmc-uk.org/professional-standards/the-professional-standards/good-medical-practice
